# Synthesis and Characterization of Boron Carbide Nanoparticles as Potential Boron-Rich Therapeutic Carriers

**DOI:** 10.3390/ma16196534

**Published:** 2023-10-02

**Authors:** Dawid Kozień, Paulina Żeliszewska, Bożena Szermer-Olearnik, Zbigniew Adamczyk, Anna Wróblewska, Agnieszka Szczygieł, Katarzyna Węgierek-Ciura, Jagoda Mierzejewska, Elżbieta Pajtasz-Piasecka, Tomasz Tokarski, Grzegorz Cios, Stanisław Cudziło, Zbigniew Pędzich

**Affiliations:** 1 Department of Ceramics and Refractories, Faculty of Materials Science and Ceramics, AGH University of Krakow, Mickiewicza, 30-059 Krakow, Poland; pedzich@agh.edu.pl; 2 Jerzy Haber Institute of Catalysis and Surface Chemistry Polish Academy of Sciences, 30-239 Krakow, Poland; zbigniew.adamczyk@ikifp.edu.pl; 3 Hirszfeld Institute of Immunology and Experimental Therapy, Polish Academy of Sciences, 53-114 Wrocław, Poland; bozena.szermer-olearnik@hirszfeld.pl (B.S.-O.); anna.wroblewska@hirszfeld.pl (A.W.); agnieszka.szczygiel@hirszfeld.pl (A.S.); katarzyna.wegierek@hirszfeld.pl (K.W.-C.); jagoda.mierzejewska@hirszfeld.pl (J.M.); elzbieta.pajtasz-piasecka@hirszfeld.pl (E.P.-P.); 4 Academic Centre for Materials and Nanotechnology, AGH University of Krakow, Mickiewicza 30, 30-059 Krakow, Poland; tokarski@agh.edu.pl (T.T.); ciosu@agh.edu.pl (G.C.); 5 Faculty of Advanced Technologies and Chemistry, Military University of Technology, Gen. Sylwestra Kaliskiego 2 Street, 00-908 Warsaw, Poland; stanislaw.cudzilo@wat.edu.pl

**Keywords:** boron carbide, nanopowder, Boron Neutron Capture Therapy (BNCT)

## Abstract

Boron carbide is one of the hardest materials in the world which can be synthesized by various methods. The most common one is a carbothermic or magnesiothermic reduction of B_2_O_3_ performed at high temperatures, where the obtained powder still requires grinding and purification. The goal of this research is to present the possibility of synthesizing B_4_C nanoparticles from elements via vapor deposition and modifying the morphology of the obtained powders, particularly those synthesized at high temperatures. B_4_C nanoparticles were synthesized in the process of direct synthesis from boron and carbon powders heated at the temperature of 1650 °C for 2 h under argon and characterized by using scanning electron microscopy, transmission electron microscopy, atomic force microscopy, X-ray diffraction analysis, and dynamic light scattering measurements. The physicochemical characteristics of B_4_C nanoparticles were determined, including the diffusion coefficients, hydrodynamic diameter, electrophoretic mobilities, and zeta potentials. An evaluation of the obtained B_4_C nanoparticles was performed on several human and mouse cell lines, showing the relation between the cytotoxicity effect and the size of the synthesized nanoparticles. Assessing the suitability of the synthesized B_4_C for further modifications in terms of its applicability in boron neutron capture therapy was the overarching goal of this research.

## 1. Introduction

Boron carbide is characterized by a high melting point [1], extreme hardness, relatively low density, a high Young’s modulus, and high chemical resistance [2]. Such properties are favorable for boron carbide applications as components resistant to abrasion in ball mills, nozzles, parts of machinery and equipment, or as parts of anti-ballistic armor [3]. Due to its high cross-section, boron carbide is used as an absorbing and screening material in the nuclear industry [1,2,3,4,5,6,7]. An additional interesting application of B_4_C may be its use in medicine as a boron delivery compound in boron neutron capture therapy (BNCT) [8,9,10].

Boron carbide has a large number of phases: B_6_C [11], B_2_C_2_ [12], BC [13], B_3_C, B_7_C, B_12_C, B_50_C_2_, B_13_C_3_ [14], and B_13_C_2_ [15]. Many research approaches have proposed BnC solid solutions, where n is in the different ranges of 24–2.57, 11.5–4, 6–5–4, 9–4, and 10.1–4. The rhombohedral (or hexagonal) B_4_C with the space group (R3m) has the most stable crystal structure [16]. The phase of the high solubility limit of carbon in boron carbide is claimed to be the boron carbide with phase composition B_13_C_2_ (B_4_C) [17,18].

Boron carbide (B_4_C) can be synthesized by various methods. The commercial method of B_4_C synthesis is a carbothermal or magnesiothermic reduction [19] of boric acid at a temperature over 2000 °C, but the powders thus obtained require grinding and purification. Due to the high value of free enthalpy of the boron carbide synthesis and high adiabatic temperature, the powder bed is diluted with B_2_O_3_, Mg, MgO, or salts such as chlorides of sodium, potassium, or magnesium. The powders, after the process of SHS, are strongly aggregated and need to have their co-products removed, usually through multiple leaching [4,8]. The low-temperature synthesis from a mixture of boric acid and glycerol calcined yields a boron carbide powder with an average grain size bigger than 1.1 μm and a residual carbon content. Other alternative sources of carbon in this method can be glycerine, polyols, phenol-liveries, citric acid, and other polymer precursors [7,8,9,10,11]. Recent research indicates that boric acid [20,21,22,23,24,25] can be easily condensed with a hydroxyl group, enhancing the esterification (B-O-C) and yielding a precursor with fine homogenous dispersion [26].

Other methods of synthesizing B_4_C are currently proposed by scientists, e.g., combining synthesis from initial compaction by pulsed electric current sintering. However, due to their low efficiency, such methods are not used in industry. The approximate 15–40 nm grain size of boron carbide can be obtained by autoclave synthesis. However, hydrothermal synthesis does not allow for obtaining non-agglomerated and aggregated particles. From amorphous boron powder and liquid CCl_4_ using lithium metals as a reducing agent, a pure nanopowder of boron carbide was synthesized from elements with the use of an amorphous carbon [27] form, i.e., soot. The obtained nanopowder contained impurities in the form of chlorides, making it inapplicable in medicine. The proposed synthesis method in the patent [1] allows one to obtain pure boron carbide with a particle size below 650 nm after synthesis in relation to commercial methods where the particle size is above 1 µm.

Major limitations in anticancer therapy are drug toxicity or rapidly developing drug resistance, a late diagnosis of quickly developed cancers, and their location. One of the promising therapies for the treatment of hard-to-reach tumors is boron neutron capture therapy (BNCT) [28]. The basis of BNCT is the “introduction” of boron compounds into tumor cells and their irradiation with low-energy epithermal neutrons. As a result, the stable isotope of boron-10 breaks down and produces high-energy alpha particles and recoiling lithium-7 nuclei [9]. Due to radiation-induced damage during the decay of nuclei ^10^B, a “therapeutic” range, comparable to the cell diameter, is postulated to be safe for healthy tissue surrounding cancer. The currently used compounds, BPA and BSH, are not evenly distributed inside the tumor cells, and therefore the boron concentration is unequal. Consequently, some cancer cells remain intact after irradiation [29]. The development studies on boron carriers led to the formation of BPA-f (boronophenylalanine complex with fructose) [30]—a molecule able to introduce boron atoms into the cell via cell membrane penetration. The fructose functionalization of BPA boosts the specific delivery of the boron carrier to neoplastic cells. The application of boron carbide in medicine is a relatively new approach, especially in anticancer therapy. One of the reported attempts to use boron carbide in experimental therapy was the treatment of oral squamous cell carcinoma xenografts in a mouse model [31]. Another example indicated that murine EL4 thymoma cells and B16 F10 melanoma cells loaded with functionalized boron carbide nanoparticles before neutron irradiation were able to inhibit the proliferation capacity of the untreated adjacent cells in an in vitro study [32]. Yet another example demonstrated the B16-OVA tumor cells incubated with boron nanoparticles administered subcutaneously to C57BL/6J mice and then irradiated with neutrons. The study revealed tumor growth retardation in contrast to the untreated control mice [33]. Similarly, a Japanese group that conducted research on the internalization of submicrometric boron carbide particles into cutaneous melanoma cells via surface coupling with transferrin proved that the internalization of large boron-rich particles was enough to achieve a therapeutic effect [28].

The aim of this research was to synthesize nanosized boron carbide powders with the use of amorphous soot as a source of carbon and amorphous boron as a source of boron. We proposed the synthesis mechanism of boron carbide via vapor deposition. The presented method is simple and neither time- nor energy-consuming. The results presented in the work and the patent allow one to obtain nanometric boron carbide with a purity of more than 99%. To confirm the potential in anticancer therapy of the obtained compounds, we performed a detailed physicochemical characteristics and cytotoxic biological activity evaluation.

## 2. Materials and Methods

### 2.1. Synthesis and Preparation of Boron Carbide Nanoparticles

The boron (Fluka 15580) and soot (Tujmazy P-803) powders were used as reagents. The synthesis of boron carbide powder was carried out according to the following procedure: first, the boron powder was placed at the bottom of a graphite crucible as a loose bed and the boron layer was carefully covered with a layer of soot. Each sample was placed in a graphite crucible and heat-treated at 1400 °C to 1700 °C, with a step of 50 °C, under the argon flow with a 10 °C/ min heating rate for 2 h.

The boron carbide powder was suspended in ultrapure water obtained with the Milli-Q Elix & Simplicity 185 purification system (Merck Millipore, Burlingtone, MA, USA). The B_4_C suspension was purified from impurities after the synthesis process using a stirred membrane filtration cell with a regenerated cellulose membrane (Millipore, NMWL: 100 Kda). When the electric conductivity of the suspension reached a minimum value of c.a. 15 μS/cm, the washing procedure was ended. The high precision densitometer DMA 5000 M (Anton Paar, Graz, Austria) was used to determine the weight concentration of the B_4_C suspension. The diffusion coefficients of B_4_C particles (fraction I and II) were determined by dynamic light scattering (DLS) using the Zetasizer Nano ZS instrument from Malvern (Malvern, UK). These data were converted to the molecule hydrodynamic diameter d*_H_* using the Stokes–Einstein relationship [34]:

(1) $$d_{H}=\frac{kT}{3\pi \eta D}$$

where *k* is the Boltzmann constant, *T* is the absolute temperature, η is the dynamic viscosity of the electrolyte, and *D* is the diffusion coefficient of the molecule derived from DLS.

The LDV technique (laser Doppler velocimetry) was used to determine the electrophoretic mobility of bare B_4_C particles $\mu_{e}$. Next, the zeta potential (ζ) of particles was calculated using Henry’s equation:

(2) $$\zeta =\frac{\eta }{\varepsilon f\left(\kappa d_{H}\right)}\mu_{e}$$

where ε is the electric permittivity of the solution and $f\left(\kappa d_{H}\right)$ is the Henry function.

For qualitative and quantitative phase analyses of the products, X’Pert PanAnalitycal Pro (Malvern Panalytical, Malvern, UK) was used. The Scherrer formula was used to calculate the crystallite size and the Rietveld analysis was used to define the qualitative analysis of the obtained powders. The morphology of reagents and products was characterized by SEM (Nova NanoSEM 200, FEI Company, Hillsboro, OR, USA) and HITACHI SU-70 (Tokyo, Japan) as well as a transmission electron microscope (TEM) (JEM 1011, Jeol, Tokyo, Japan).

### 2.2. Biological Activity Evaluation of the Obtained Boron Carbide Nanoparticles

#### 2.2.1. Cell Lines

Mouse cell lines: RAW 264.7 and J774A.1 cells of the mouse macrophage cell line received from ATCC (Manassas, VA, USA) (TIB-71^TM^; TIB-67^TM^) and NIH/3T3 cells of the mouse fibroblast cell line obtained from ATCC (CRL-1658^TM^) were cultured on DMEM (ATCC) supplemented with 100 U/mL penicillin, 100 mg/mL streptomycin, and 10% fetal bovine serum (FBS for RAW264.7 and J774A.1 cells) or 10% fetal calf serum (for NIH/3T3 cells) (all from Sigma-Aldrich, St. Louis, MO, USA). JAWS II cells of the immature mouse dendritic cell line received from ATCC (CRL-11904^TM^) were maintained in a 1:1 mixture of MEM-α (Gibco, Billings, MT, USA) and RPMI 1640 (Gibco) supplemented with 100 U/mL penicillin, 100 mg/mL streptomycin, 0.5 mM sodium pyruvate, 10% FBS (all from Sigma-Aldrich), and 5 ng/mL recombinant murine GM-CSF (ImmunoTools, Friesoythe, Germany). MC38/0 cells of the in vivo growing murine colon carcinoma from the Tumor Bank of the TNO Radiobiology Institute, Rijswijk, Holland, were adapted to in vitro conditions as described by Pajtasz-Piasecka et al. [35]. The culture of MC38/0 (here named MC38) was maintained in RPMI 1640 medium (Gibco) supplemented with 100 U/mL penicillin, 100 mg/mL streptomycin, 1 mM sodium pyruvate, 0.05 mM 2-mercaptoethanol, and 5% FBS (all from Sigma-Aldrich).

Human cell lines: THP-1 cells of the human monocytic cell line received from ATCC (TIB-202^TM^) were cultured on RPMI 1640 (Gibco) supplemented with 100 U/mL penicillin, 100 mg/mL streptomycin, 1 mM sodium pyruvate, 0.05 mM 2-mercaptoethanol, and 10% FBS (all from Sigma-Aldrich). HT-29 cells of the human colon adenocarcinoma cell line received from ATCC (HTB-38^TM^) were maintained in DMEM (ATCC) supplemented with 100 U/mL penicillin, 100 mg/mL streptomycin, and 10% FBS (all from Sigma-Aldrich). T98G cells of the human glioblastoma cell line obtained from ATCC (CRL-1690^TM^) were cultured on EMEM (ATCC) supplemented with 100 U/mL penicillin, 100 mg/mL streptomycin, and 10% FBS (all from Sigma-Aldrich). All the cell cultures were grown at 37 °C, with 95% humidity and 5% CO_2_.

The maintenance of cell lines and performance of in vitro assays were carried out at the Hirszfeld Institute of Immunology and Experimental Therapy, Polish Academy of Sciences, Wrocław, Poland.

#### 2.2.2. MTT Assay

The RAW 264.7, J774A.1, NIH/3T3, MC38/0, HT-29, and T98G cells were placed in 96-well plates (Corning Costar, Corning, NY, USA) at a density of 5 × 10^3^ cells/well and JAWS II and THP-1 cells at a density of 1 × 10^4^ cells/well. After 24 h, the aqueous suspensions of boron carbide preparations (B_4_C 1, B_4_C 2) and BPA ((L)-4-dihydroxy-borylphenylalanine) (Sigma-Aldrich) were diluted in the culture medium (appropriate for each cell) to neutralize the pH and were added at various concentrations (in the range from 0.1 to 400 µg/mL) to the plates with the cells. The cells were incubated with the compounds for 72 h. Next, the MTT dye (3-(4.5-dimethylthiazol-2-yl)-2.5-diphenyltetrazolium bromide; 5 mg/mL) (Sigma-Aldrich) was added for 4 h. After this time, the cells were lysed overnight in a lysis buffer (N,N-dimethylmethanamide, sodium dodecyl sulfate, and water) at 37 °C. The absorbance value was recorded at 570 nm using a Thermo Labsystems Multiskan RC microplate reader (Thermo Fisher Scientific Inc., Waltham, MA, USA) with Genesis Lite 3.05 Software (Thermo Life Sciences). The IC_50_ values were calculated using GraphPad Prism 9 software (GraphPad Software, La Jolla, CA, USA).

#### 2.2.3. Statistical Analysis

All the data were analyzed using GraphPad Prism 9 software (GraphPad Software, Inc.). In comparable analyses of the two groups, the statistical differences were calculated using the unpaired *t*-test. All statistically significant differences are presented in the graphs; otherwise, the differences were not significant.

## 3. Results

### 3.1. Obtaining and Analyzing the Surface of Boron Carbide Nanoparticles

**Figure 1:**
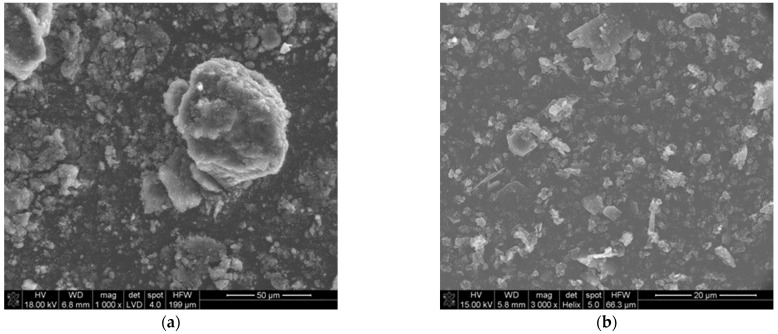
SEM images of reagent powders: (**a**) amorphous soot, (**b**) amorphous boron powder.

The morphology of the precursor is shown in Figure 1. Figure 1a shows the morphology of the amorphous soot. It consists of small particles (400–800 nm) strongly aggregated (10–20 μm). The amorphous boron has an average particle size of ~800 nm (Figure 1b).

**Figure 2:**
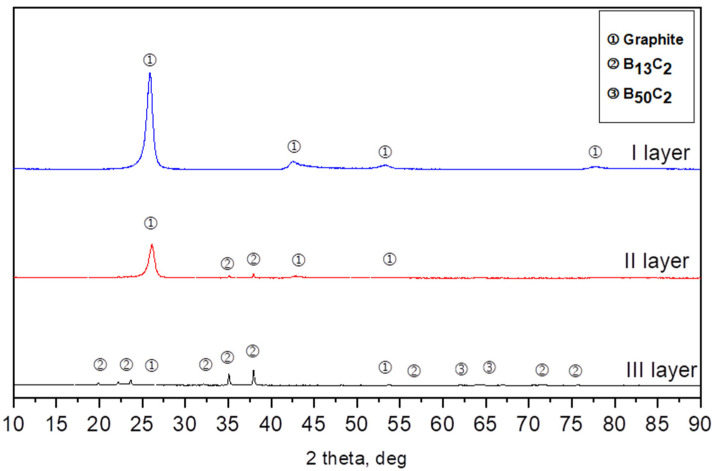
Example of XRD patterns of the synthesis from elements at 1700 °C.

Three types of layers differing in color were distinguished optically in the bed after synthesis. Therefore, each layer was separately subjected to the X-ray diffraction analysis. Layer 1 was located on the top of the crucibles; below this was layer 2, while layer 3 was located at the bottom of the crucibles. The X-ray diffraction analysis revealed that the powder from layer 1 was composed of one-phase graphite (ICSD 98-007-6767), obtained after being synthesized from soot. Powder from layer 2 was composed of a two-phase rhombohedral boron carbide with a stoichiometry close to B_13_C_2_ (ICSD 98-061-2568) and a small amount of graphite. The obtained powders from layer 3 were composed of three phases: the rhombohedral phase of boron carbide with a stoichiometry close to B_13_C_2_, the tetragonal B_48_(B_2_C_2_) phase, and a small amount of graphite-like carbon less than 1%. At each temperature, we obtained three layers after boron carbide synthesis. The biggest difference is the contents between the rhombohedral phase of boron carbide B_13_C_2_ and the tetragonal B_48_(B_2_C_2_) (ICSD 98-000-9734) phase (Figure 2).

**Figure 3:**
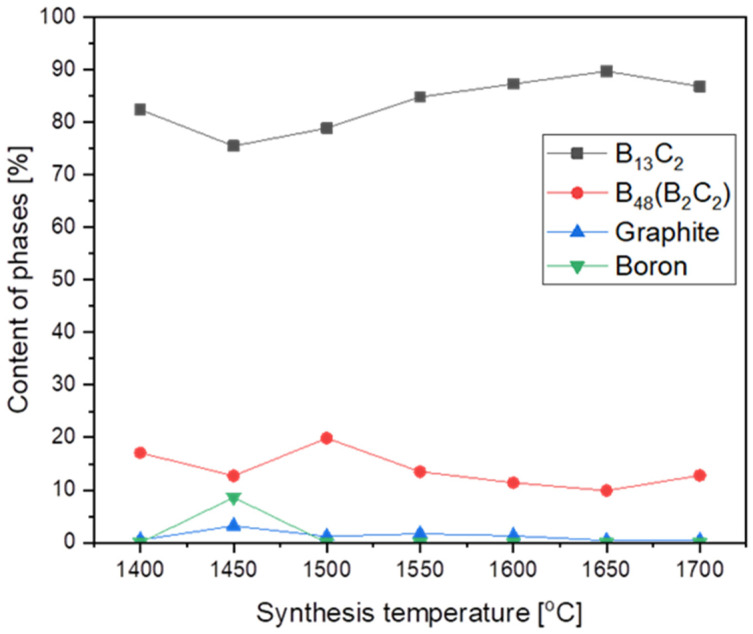
The phase composition of synthesized products vs. synthesis temperature.

The highest structure stability, and hence the highest melting point, demonstrated the boron carbide with B_13_C_2_ stoichiometry corresponding to 13.3 at. % of carbon. As the synthesis temperature increased, the synthesis product tried to reach the highest structure and achieved this at 1650 °C (Figure 3) with the biggest phase of B_13_C_2_, with no particle growth. Already at 1700 °C, there was a significant growth of nanoparticles, from 13.4 nm at 1650 °C to 41 nm at 1700 °C. (Table 1).

**Figure 4:**
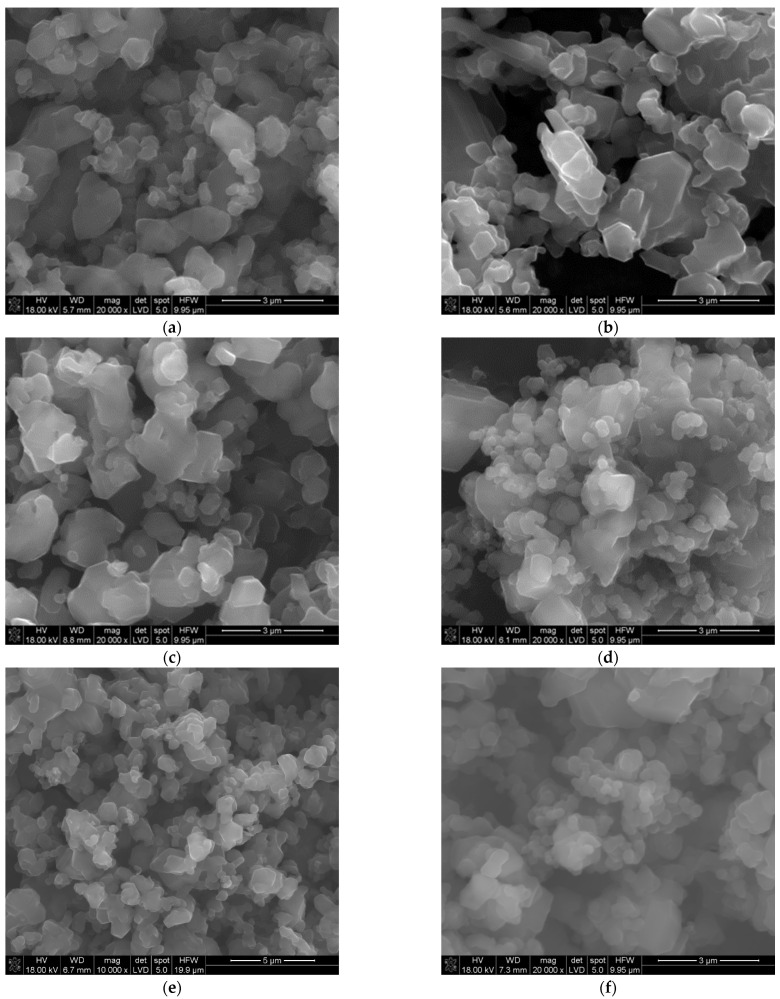
SEM morphology of the powders synthesized at different temperatures: (**a**) 1450 °C, (**b**) 1500 °C, (**c**) 1550 °C, (**d**) 1600 °C, (**e**) 1650 °C, (**f**) 1700 °C.

**Figure 5:**
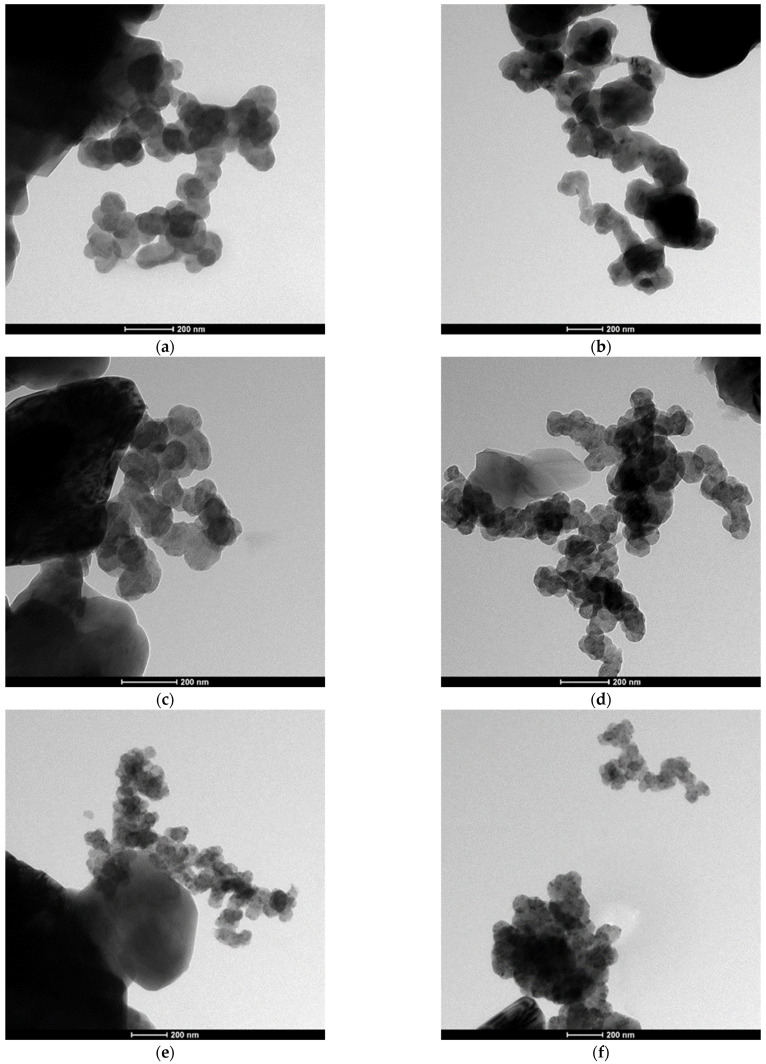
TEM morphology of the powders synthesized at different temperatures: (**a**) 1400 °C, (**b**) 1450 °C, (**c**) 1550 °C, (**d**) 1600 °C, (**e**) 1650 °C, (**f**) 1700 °C.

**Figure 6:**
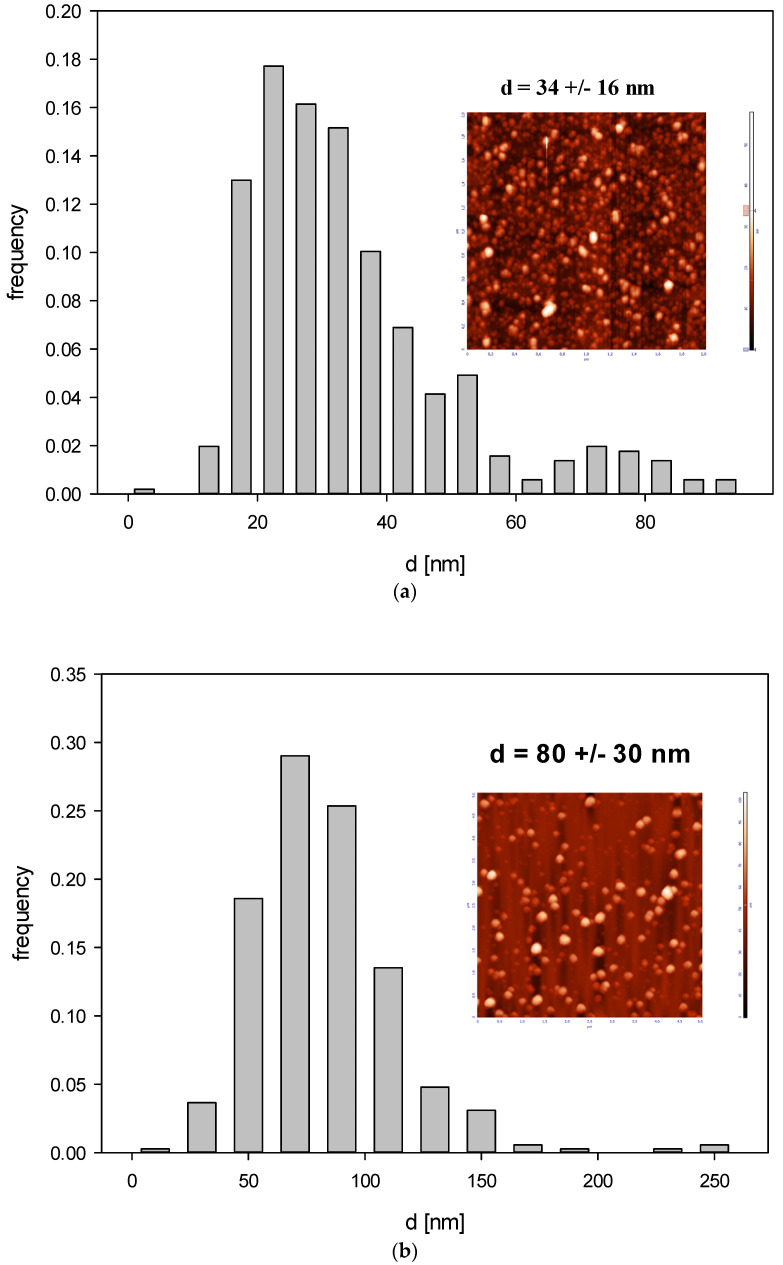
Histogram of B_4_C particle size distribution (part (**a**)—fraction 1, part (**b**)—fraction 2) derived by AFM imaging. The inset shows an AFM image of the nanoparticles.

The SEM and TEM observations of the synthesized powders (Figure 4 and Figure 5) supported crystallite morphology determined by the XRD analysis (Figure 6) and also revealed strong aggregation of the primary particles. The SEM and TEM microphotographs (Figure 4 and Figure 5) confirmed the narrow particle size distribution.

The applied research methods as well as the XRD, SEM, and TEM analyses of the obtained powders allowed us to select the powder for further processing. The detailed preparation of boron carbide suspensions is described in the patent [1]. The essence of the method of obtaining boron carbide nanoparticles is the process of direct synthesis from boron and carbon powders heated at the temperature of 1650 °C for 2 h under argon flow. The thus obtained powder is characterized by the highest content of boron (at the level of 99%), with the smallest crystallite sizes (Table 1) and an average size of the agglomerates of about 650 nm. The product bed was obtained, according to the kinetics of the reaction, in the form of three distinguishable layers; the top two layers fell off and/or were recycled as raw material for re-synthesized boron carbide, and the lowest layer at the bottom of the crucible, distinguished by a light gray color, showed the greatest brevity and hardness. The synthesis was performed 10 times and the obtained gray powders were mixed. The weight of the further-treated powder was 1 kg. The resulting powder was subjected to intensive grinding with steel elements in isopropanol for 24–48 h and then exposed to chemical etching to flush ion impurities iron from milling using, sequentially, the concentrated HCl, the concentrated HNO_3_, and the concentrated HCl again. The resulting powder was surrendered by repeated rinsing with distilled water until the pH of the suspension reached a value ranging from 6.5–7.5, and finally the suspension was subjected to intensive centrifugation. The use of ultracentrifugation allowed us to select two suspensions. One suspension at a speed of 9000 to 12,000 rpm, with a spin time ranging from 1 h to 4 h, produced rounded boron carbide, described further as fraction 1, and the second fraction (fraction 2) was obtained by a spin speed of 5000 to 8000.

The physicochemical characteristics of B_4_C particles were measured by determining the diffusion coefficients, hydrodynamic diameter, electrophoretic mobilities, and zeta potentials.

The particle size distribution was determined using AFM imaging in the air using the NT-MDT Solver BIO device with the SMENA SFC050L scanning head. All the measurements were performed in a semi-contact mode by using high-resolution silicon probes (NT-MDT ETALON probes, HA NC series, polysilicon cantilevers with resonance frequencies 240 kHz ± 10% or 140 kHz ± 10%, force constants 9.5 N/m ± 20% or 4.4 N/m ± 20%, respectively; the typical curvature radius of the tip was 10 nm and the cone angle was less than 20°). The images were determined within the scan area suitable for the tested samples. All the images were flattened using an algorithm provided by the instrument.

The topographical analysis of the particle layer images acquired by AFM was carried out by determining the height profiles and calculating the root mean square roughness (the interface width) using specialized software. These results were interpreted in terms of theoretical results derived by numerical integration over 2D domains, which enabled the quantitative determination of the particle shape, thickness, and heterogeneity of the particle layer. The root mean square roughness of an interface is defined as [36]:

(3) $$rms_{}^{2}=\frac{1}{S}\int_{s}{\left(h\left(r_{s}\right)-\bar{h}\right)}^{2}dr_{s}=\frac{1}{S_{}}\int_{s}h^{2}\left(r_{s}\right)-{\bar{h}}^{2}$$

where *S* is the geometrical area of the AFM image, *h*(r_s_) is the local height of the surface profile measured relative to the reference value *h*_0_, and $\bar{h}$ is the average height of the particle layer given by the formula:

(4) $$\bar{h}=\frac{1}{S_{}}\int_{s}h\left(r_{s}\right)dr_{s}$$

r_s_ is the surface position vector.

Equation (3) can be analytically evaluated for particles of a regular shape. For example, in the case of ellipsoidal particles, their shape is defined by the governing equation:

(5) $$\frac{x^{2}}{a^{2}}+\frac{y^{2}}{b^{2}}+\frac{z^{2}}{c^{2}}=1$$

where *a*, *b*, and *c* are the semi-axes of the ellipsoid. If *a = b*, Equation (5) defines an oblate spheroid, and in the case of *b = c*, Equation (5) defines a prolate spheroid. Obviously, for *a = b = c,* one obtains *a* spherical shape.

Considering the constitutive Equation (5), one derives after integration the following expressions for the average height and the surface rms:

(6) $$\begin{array}{l} \bar{h_{1}}=\frac{5}{3}R_{c}^{}\\ rms={\left[\frac{17}{24}\Theta_{}\left(1-\frac{50}{51}\Theta_{}\right)\right]}^{1/2}d_{p} \end{array}$$

where Θ= π abN is the dimensionless coverage of particles, *N* is their surface concentration conveniently expressed as the number of particles per one square micrometer, and $d_{p}=c$ is the particle thickness equal to the shorter spheroid axis.

Thus, knowing the particle coverage in the monolayer, one can calculate the particle thickness by inverting Equation (6):

(7) $$d_{p}=1.188{\left[\Theta_{}\left(1-0.980\Theta_{}\right)\right]}_{}^{-1/2}rms$$

It should be mentioned that the particle thickness cannot be derived from the DLS measurements of the diffusion coefficient where the hydrodynamic diameter of particles is calculated using Equation (2).

Accordingly, the size of B_4_C nanoparticles derived from AFM imaging (see Figure 6) was 32 ± 10 nm for fraction 1 and 80 ± 30 nm for fraction 2 and the diffusion coefficient derived from DLS for pH range 2.5 –11 and ionic strength 10^−3^–10^−2^ M was equal to 2.17 × 10^−7^ ± 1.18 × 10^−7^ cm^2^ s^−1^ and 2.93 × 10^−7^ ± 0.143 × 10^−7^ cm^2^ s^−1^ for fraction I and II, respectively.

Additionally, the rms for fraction 1 was equal to 4.1 nm for = 0.12, which yields 15 nm for the particle thickness. For fraction 2, the rms was equal to 17 nm for 0.07 nm Θ = 0.07, yielding 79 nm for the particle thickness. These results indicate that the shape of smaller particles (fraction 1) can be approximated by an oblate spheroid with the dimensions 32 × 32 × 15 nm, whereas the shape of larger particles (fraction 2) can be approximated by a sphere.

The results obtained by AFM agree with those derived from DLS measurements carried out for various pHs and with ionic strengths of 10^−3^ and 10^−2^ M; see Table 2 and Table 3.

**Figure 7:**
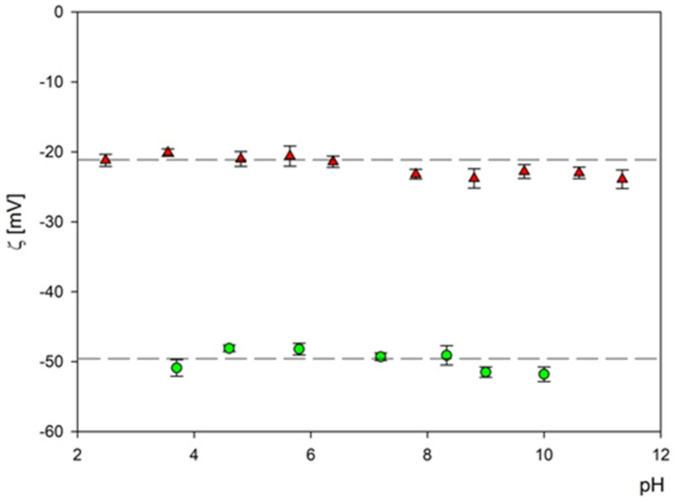
The dependence of the zeta potential of boron carbide nanoparticles on pH at an ionic strength of 10^−3^ M for fraction 1 (▲)—and fraction 2 (●). The dash lines are the linear fits of experimental data.

Subsequently, the electrophoretic mobility μϵ of particles was measured as a function of pH using the LDV method. It was confirmed that the B_4_C suspension (fractions 1 and 2) remained stable for the NaCl concentration up to 10^−2^ M and with a pH range of 2–11. The hydrodynamic diameter of the B_4_C suspension was equal to 29 ± 7 nm and 66 ± 5 nm for fraction 1 and 2 and the 10^−3^ M NaCl concentration, respectively. As can be observed with the increasing pH, the hydrodynamic diameter slightly increased for both fractions. The zeta potential of the B_4_C particles was negative and independent of pH for both fractions, as can be seen in Figure 7. For convenience, these values are summarized in Table 2 and Table 3.

**Figure 8:**
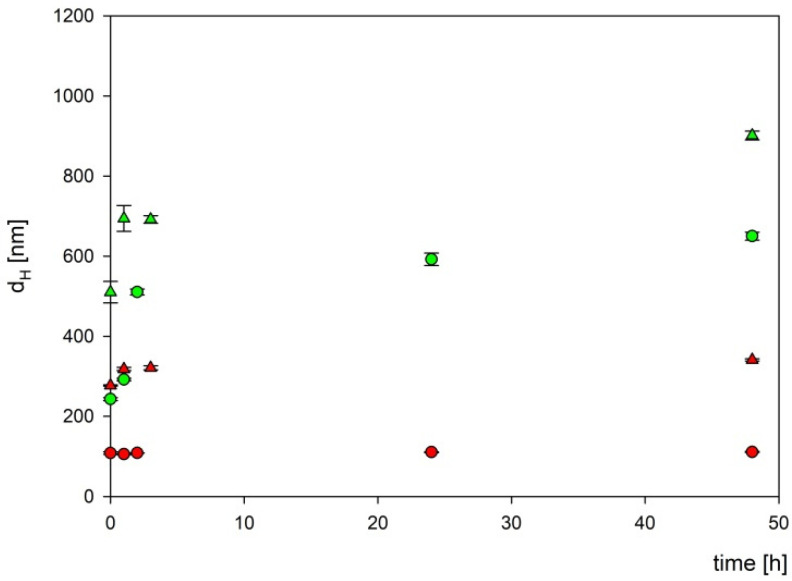
The dependencies of the hydrodynamic diameter of B_4_C nanoparticles on incubation time. The points denote experimental results obtained for fraction 1 (●), fraction 2 (▲), DMEM (red color), and RPMI (green color). The B_4_C bulk concentration was equal to 1000 mg L^−1^.

To exclude the influence of the components of the culture medium on the boron carbide nanoparticles, their stability in two different culture media appropriate for tested cell lines (DMEM and RPMI) was assessed. For this purpose, two fractions of boron carbide particles at a concentration of 1000 mg L^−1^ were mixed with two types of culture media and then incubated at 37 °C for the specified time. As can be seen in Figure 8, two fractions of B_4_C particles were stable for more than 24 h in the DMEM culture medium, while in the RPMI medium, the aggregation appeared after 3 h of incubation.

### 3.2. Analysis of the Cytotoxic Effect of Obtained Boron Carbide Nanoparticles

**Figure 9:**
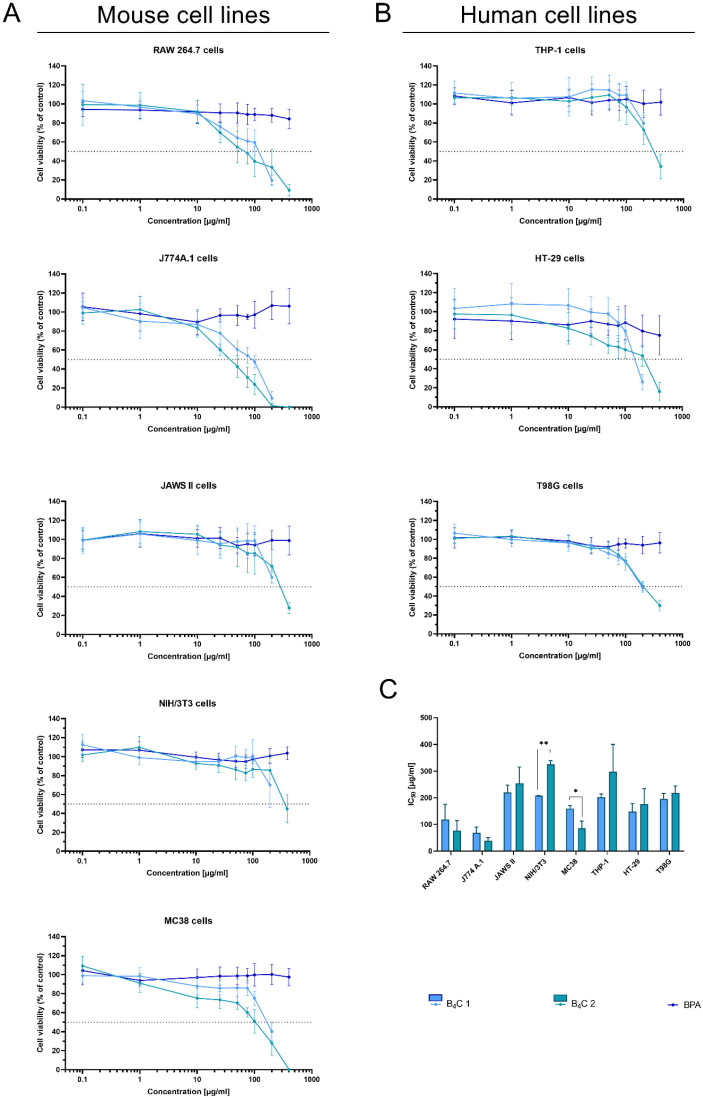
Effect of boron carbide preparations on cell proliferation. The viability of (**A**) mouse cells (RAW 264.7, J774A.1, JAWS II, NIH/3T3, MC38) and (**B**) human cells (THP-1, HT-29, T98G) after 72 h of exposure to boron carbide compounds (B_4_C 1 and B_4_C 2) and BPA was assessed using the MTT assay. Graphs represent the percentage of viable cells relative to control cells (control = 100%). (**C**) Concentrations of B_4_C 1 and B_4_C 2 causing 50% inhibition of cell proliferation (IC_50_) calculated for each cell line. Results are expressed as means ± SD calculated for three independent experiments performed in triplicate. The differences between groups were calculated using multiple unpaired t-tests (* *p* < 0.05; ** *p* < 0.01).

The MTT assay is one of the most frequently performed tests evaluating cell viability in cultures in vitro. It determines the compound’s effectiveness in inhibiting biological cellular functions, defined as half the maximum inhibitory concentration (IC_50_). Therefore, the MTT assay was employed to estimate the cytotoxicity of synthesized boron carbide compounds (fraction 1 and fraction 2). Due to its wide range of applications in clinical trials of BNCT, boronophenylalanine (BPA) was used as a control. The effect of the nanoparticles was analyzed on human and mouse cell lines represented by cells with different functions, such as phagocytic cell lines RAW 264.7, J774A.1, JAWS II, and THP-1, normal cell line NIH/3T3, and tumor cell lines MC38, HT-29, and T98G (Figure 9A,B).

As expected, BPA showed no cytotoxic activity in the range of the tested concentrations, similarly to the other report [37]. After 72 h of cell exposure to the boron carbides, the different cell sensitivity was observed depending on the cell types. The mouse macrophages, RAW 264.7 and the J774A.1 cells, were the most sensitive to the tested compounds. The observed effect depended on the compound concentration and was most likely associated with the natural predisposition of phagocytic cells to nanoparticle uptake and its internalization. For both types of cells, the IC_50_ ranged between 50 and 100 ug/mL of compounds. However, B_4_C fraction 2 revealed higher toxicity towards applied macrophages than fraction 1 (preparation containing smaller boron carbide particles). This observation suggests a relationship between particle size and shape and cytotoxicity.

In contrast to these cells, the toxicity of the compounds towards other (both non-malignant and tumor) cells was significantly lower (the IC_50_ values for JAWS II or NIH/3T3 were two- or three-fold higher compared to the macrophages of murine cell lines). Moreover, the NIH/3T3 cells of the normal fibroblasts’ line turned out to be the least sensitive to boron carbide, especially to fraction 2.

The tumor cells turned out to be less sensitive to the tested compounds than the murine macrophages but more sensitive than the human monocyte-like THP-1 cells. Among the tested tumor lines, MC38 was the most sensitive to boron carbide, but this may be due to the fact that aggregation of the compound occurs in the RPMI medium used for its cultivation, which may be correlated with a size-dependent toxicity effect. Of note, in contrast to BPA [38], boron carbides present good stability in water, which enhances the uptake of their small-particle fraction, especially by non-phagocytic cells. This B_4_C property can also facilitate its wide spreading in the biological environment and increase the effective delivery into the tumor site.

The physicochemical analysis of the obtained boron compounds correlated with their behavior in culture media, and the relation between cell reactivity and the size of boron carbide nanoparticles will be extremely helpful in further studies on the biological properties of both fractions and their suitability for the design of functionalized particles dedicated to BNCT.

## 4. Conclusions

The obtained results allow for drawing several conclusions which are important in terms of further modifications of the obtained boron compounds in order to use them in biomedical application such as targeted BNCT:

- In each obtained powder, the dominant phases of boron carbide were rhombohedral B_13_C_2_ and tetragonal B_48_(C_2_B_2_).
- The results suggest that the mechanism of boron carbide synthesis by the reaction between boron vapor and carbon cannot be excluded. However, this is not the only possible mechanism of boron transport.
- Boron carbides present good dispersion in water, which enhances uptake of their small-particle fraction, also by non-phagocytic cells. It can also facilitate their wide spreading in the biological environment and increase the effective delivery into the tumor site.
- The obtained boron carbide, after further surface modifications of the compound that will increase specificity for target cells, has the potential to be used as a new boron carrier in BNCT.

## 5. Patents

Patent application to the Patent Office of the Republic of Poland entitled “Synthesis and preparation of boron carbide (B_13_C_2_) nanoparticles” with the number: P.440310 [WIPO ST 10/C PL440310].

## Figures and Tables

**Table 1:** The particle size of powders synthesized at different temperatures measured by Scherrer formula for synthesis and grey layer closest to the layer of boron.

Synthesis Temperature [°C]	B_13_C_2_-d_XRD_ [nm]	B_48_(B_2_C_2_)-d_XRD_ [nm]
1400	11.5	7.8
1450	10.7	8
1500	10.4	7.8
1550	12.4	10.2
1600	16.2	12.8
1650	13.4	12.6
1700	41	7.2

**Table 2:** Zeta potential, electrokinetic charge density, the specific surface area of the B_4_C (fraction 1) for various pHs and ionic strengths equal to 10^−3^ M NaCl.

10^−3^ M NaCl										
pH	2.48	3.55	4.08	5.64	6.38	7.8	8.8	9.66	10.6	11.34
µe [µm cm V^−1^s^−1^]	−1.66	−1.58	−1.65	−1.61	−1.68	−1.81	−1.87	−1.77	−1.80	−1.87
ζ [mV]	−21.2	−20.1	−21	−20.6	−21.4	−23.2	−23.8	−22.8	−23	−23.9
ζ Henry [mV]	−28.9	−28.7	−30.0	−29.5	−30.1	−32.5	−33.6	−31.8	−32.2	−33.4
d_H_ [nm] DLS by number	31 ± 8	29 ± 7	29 ± 7	28 ± 6	43 ± 7	42 ± 8	42 ± 9	42 ± 6	41 ± 9	45 ± 10
Charge Density [e nm^2^]	−0.0281	−0.0154	−0.0149	−0.0140	−0.0142	−0.0165	−0.0167	−0.0154	−0.0157	−0.0165
Sg [nm^2^] ×10^3^	3.02	2.64	2.64	2.46	5.81	5.54	5.54	5.54	5.28	6.36

**Table 3:** Zeta potential, electrokinetic charge density, the specific surface area of the B_4_C (fraction 2) for various pHs and ionic strengths equal to 10^−3^ M NaCl.

10^−3^ M NaCl							
pH	3.7	4.6	5.8	7.2	8.33	9.02	10
µe [µm cm V^−1^s^−1^]	−3.99	−3.77	−3.78	−3.86	−3.85	−4.04	−4.06
ζ [mV]	−50.9	−48.1	−48.2	−49.3	−49.1	−51.5	−51.8
ζ Henry [mV]	−56	−55	−57	−58	−57	−60	−61
d_H_ [nm] DLS by number	66 ± 5	64 ± 12	64 ± 10	66 ± 8	70 ± 6	69 ± 5	64 ± 8
Charge Density [e nm^2^]	−0.0335	−0. 0302	−0.0314	−0.0321	−0.0314	−0.0337	−0.0345
Sg [nm^2^] ×10^4^	1.37	1.29	1.29	1.37	1.54	1.50	1.29

## Data Availability

Not applicable.
